# Interspecific differences and commonalities in maternity roosting by tree cavity-roosting bats over a maternity season in a timber production landscape

**DOI:** 10.1371/journal.pone.0194429

**Published:** 2018-03-15

**Authors:** Niels Rueegger, Brad Law, Ross Goldingay

**Affiliations:** 1 School of Environment, Science and Engineering, Southern Cross University, Lismore, New South Wales, Australia; 2 Forest Science, New South Wales Primary Industries, Parramatta, New South Wales, Australia; University of Reunion Island, RÉUNION

## Abstract

Understanding maternity roost requirements is fundamental to guide timber production forest management given such roosts are vital to sustain bat populations. We tracked lactating females of three tree cavity-roosting species: Gould's long-eared bat (*Nyctophilus gouldi*) (n = 7), eastern broad-nosed bat (*Scotorepens orion*) (n = 6) and little forest bat (*Vespadelus vulturnus*) (n = 25), over five weeks in young (predominately <5 years old) forest regenerating from heavy timber harvest in southeast Australia. We aimed to investigate interspecific maternity roost selection in a regenerating landscape and by doing so, increase our understanding of the three species’ roost ecology. Sixteen *V*. *vulturnus*, 15 *N*. *gouldi* and six *S*. *orion* unique maternity roost trees were located. Bats displayed a degree of maternity roost selection plasticity, however, interspecific differences were found. *Nyctophilus gouldi* roosted selectively in retained riparian buffers, in trees of high senescence and switched roosts every day. *Vespadelus vulturnus* roosted in logged areas and displayed high roost site fidelity, with one roost used for 33 consecutive days. *Scotorepens orion* selected large live trees of low senescence. The preliminary data for this species suggests that females roost most days in ‘primary’ roosts but display a roost switching behaviour conforming to the fission-fusion model. Dead trees were identified to be important for both *N*. *gouldi* and *V*. *vulturnus*. Historical and recent logging at our study area drastically reduced cavity-bearing tree density to 1.4 trees per hectare in the logging zones (outside of exclusion areas), potentially limiting local populations of tree cavity-roosting bats and other cavity-dependent wildlife. Our data demonstrate that forest management must consider a range of maternity roost requirements to accommodate differences among species and highlight the importance of exclusion areas for roost habitat. We propose that an expanded ‘retention forestry’ approach should be implemented in logged areas that includes in-perpetuity forest patch retention to increase habitat complexity and continuity.

## Introduction

Given a large proportion of a bat’s life is spent in roosts, roost sites are fundamental for survival and the quality of available roosts have the potential to influence fitness of individuals [1–4]. The management of maternity roosts is particularly crucial as this roost type is a key resource for sustaining viable populations [5] with roost characteristics being more specific than other day roosts (e.g., [5–9]). As such, in disturbed landscapes, maternity roosts have the potential to be a limiting resource (e.g., [5,10–12]).

One industry that is required to manage tree cavity-roosting bats is forestry. Forest management practices differ vastly across the world [13,14] with unregulated timber harvesting being considered a major threat to bats worldwide, including timber extraction in old-growth forest, clear felling practices and some forms of selective logging [15]. In Australia, silvicultural practices range from clear-cut methods to less intensive group or selective tree harvesting [14]. Knowledge about the ecological implications of different silvicultural practices and mitigation measures is essential for the conservation of cavity-using wildlife including bats [13,14,16] so the need for adequate cavity-bearing tree retention in commercial forests is critical (e.g., [14,17–19]).

Ongoing knowledge gaps for tree cavity-roosting bats in commercial forests include species-specific maternity roost preferences, maternity roost habitat selection, spatial maternity roost distribution, maternity roost fidelity and the effectiveness of prescribed vegetation retention measures. Previous studies have shown that riparian buffers are an important roost habitat for some bat species [14]. However, species-specific knowledge is lacking for most species including the effectiveness of retained habitat trees within logged areas, with studies reporting contrasting use of retained roost habitat (e.g., [6,16,20–23]). Furthermore, there is a distinct lack of knowledge about minimum thresholds for landscape exclusion of harvesting and suitable cavity-bearing tree thresholds [24] for bats, which likely differ among species and localities [13,25].

Longer-term patterns of roost use, such as over a maternity season, are also poorly known for bats. Radio-tracking studies on tree cavity-roosting bats have generally been short-term due to weight constraints of attaching transmitters to small animals, with a few long-term studies investigating roost re-use over multiple years [26–28]. Australia contains a high diversity of bat species with about 43 species (68%) of echolocating bats roosting in tree cavities (derived from [29]). We radio-tracked three tree cavity-roosting species, Gould's long-eared bat (*Nyctophilus gouldi*), eastern broad-nosed bat (*Scotorepens orion*) and little forest bat (*Vespadelus vulturnus*) over the first five weeks post parturition. Previous radio-tracking of lactating *N*. *gouldi* found that maternity roost habitat was predominantly in gullies and riparian corridors within dense forest cover, and that roost switching was more frequent for maternity roosts than other day roosts [30,31]. Previous radio-tracking of *V*. *vulturnus* in managed woodlands documented that dead trees and dead sections in live trees were important for roosting [32], although this was not found in an agricultural landscape, where live roost trees were more commonly used [33]. The studies on *V*. *vulturnus* did not track female bats during the maternity season and no details are known about this species’ maternity roost selection. Similarly, no data on maternity roost selection have been published for *S*. *orion*. The three species radio-tracked are considered common in south-east Australia [29] and are not listed as threatened. The species were chosen because little information is known about their long-term pattern of maternity roost use and because the species could be trapped with some reliability that ensured adequate sample sizes. This study was located in a predominantly recently harvested forest landscape in southeast Australia. The dominant silvicultural practice at the study site comprised harvesting trees in compartments (~200 ha) with the retention of habitat trees within the logged area (dispersed tree retention) and exclusion zones (predominantly in gullies along creek lines). This type of harvest results in mosaic patches of different aged regrowth forest comprising isolated emergent trees across the landscape and undisturbed vegetation predominantly along stream lines, but also connecting adjacent catchments via corridors. Our specific aims were: (1) to investigate maternity colony size and maternity roost site fidelity over a five week tracking period, (2) to investigate whether species differed in their maternity roost tree selection, and (3) to investigate whether species selected maternity roosts in specific habitat types (riparian zone, logged areas, non-riparian mature forest). We hypothesised: that bats would select roosts in areas of high tree cavity abundance (e.g., [16,34,35]), in large trees (e.g., [16,36,37]) and dead trees that were locally uncommon (e.g., [9,35,38]). In addition, we hypothesised that the larger bat species would encompass a larger roost range, that lactating females of the same species trapped at the same location would congregate at the same roost sites, and that roost switching would be frequent (e.g., [30,39]).

## Methods

### Study area

**Fig 1:**
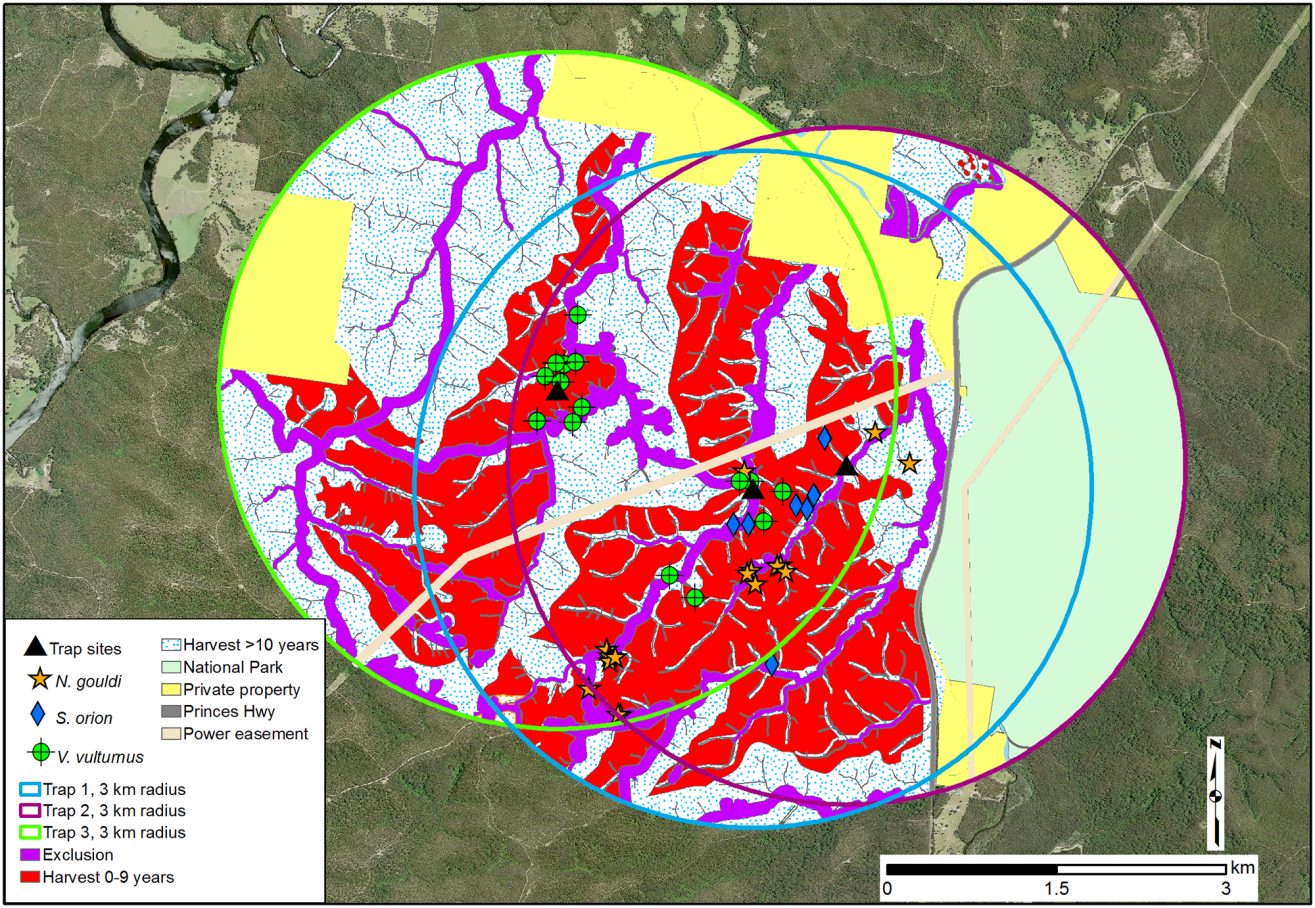
Trap locations, roost sites and habitat types within 3 km radii surrounding the trap sites. Base map of image is reprinted with permission from the New South Wales Department of Finance, Services & Innovation under a CC BY licence, original copyright Department of Finance, Services & Innovation [30/10/2017].

Bats were studied in Boyne State Forest (35°35'S; 150°14'E), south-eastern Australia across a landscape that comprised logged areas (harvest coupes) of both recently (< 10 years) and older (≥ 10 years) harvested forest stands, as well as harvesting exclusion zones predominantly made up of densely vegetated riparian buffers (Fig 1). The harvesting history immediately surrounding the trap sites was predominately < 5 years since the last harvest event. Within a 10 km radius, the harvested areas made up 82% of the State Forest land. Areas excluded from logging were predominantly gully vegetation, but also included an over-ridge connection corridor and, in the wider area, landscape exclusions for owls, as well as National Park land.

The dominant vegetation type was dry sclerophyll forest with spotted gum (*Corymbia maculata*) being the principal tree species with a mean canopy height of 22 m. Along gully-lines, temperate rainforest vegetation was present which was dominated by grey myrtle (*Backhousia myrtifolia*) and was interspersed with a sparse emergent cover of eucalypts. Three trap sites were used. Two were located over separate creek lines (Site 1 and 2) and the other was at a ridge top across a logging track (Site 3) (Fig 1).

The study area (defined as a 3 km radius surrounding the trap sites) was located within an area designated as a ‘forest regrowth zone’ for timber production. This zone has a requirement to retain up to five cavity-bearing trees ha^-1^ in logged areas plus an equivalent number of recruitment trees to that of the number of retained cavity-bearing trees. For example, if there are only two cavity-bearing trees ha^-1^ found in a current operation, only two cavity-bearing recruitment trees would need to be retained plus recruits for each of these. In addition, specific prescriptions in the study area relevant to tree cavity-roosting bats included: retention of up to five dead trees ha^-1^ (if present) in logged areas, logging exclusion in riparian buffers along the length of the stream (buffer width dependent on stream order), logging exclusion in rainforest, retention of old-growth forest areas (if present), key habitat for threatened species and retention of ‘ridge to headwater’ buffers [40]. ‘Ridge to headwater’ buffers intend to link neighbouring catchments and are prescribed as either two 40 m wide exclusion zones connecting second order streams, or one 80 m wide exclusion zone connecting third order streams for every 500 ha [40].

### Harp-trapping and radio-tracking

Bats were caught using Austbat harp traps (research equipment Faunatech, Australia) at three sites. Two were located over separate creek lines (Site 1 and 2) and the third was at a ridge top across a logging track (Site 3). Bats were radio-tracked during the maternity season from the end of November 2015 to start of January 2016 (five weeks). The start of the trapping occurred just at the cusp of post-parturition with some pregnant bats captured on the first two days (identified through the pronounced abdomen) with all subsequently trapped bats lactating (recognised through a lack of distinct belly shape, well developed nipples, expression of milk and/or bare patches around nipples).

Radio-transmitters weighed 0.31 g (Holohil Systems Ltd, Ontario, Canada) and were < 5% of the body mass for *N*. *gouldi* and *S*. *orion* and weighed about 7.4% of *V*. *vulturnus* mass, which was more than the recommended 5% by Aldridge and Brigham [41], but less than 10% recommendation by Bradbury et al. [42]. The mean weight of lactating *V*. *vulturnus* was 4.2 g, though pregnant *V*. *vulturnus* weighed 5.7 g and therefore, carried ~ 35% more body weight during pregnancy. The transmitters were glued to where the bat’s shoulder blades met using Uro-Bond IV, Urocare. Bats were radio-tracked during the day to locate their roost tree and a GPS point was obtained. Some of the roost trees were stag-watched (i.e. bats were counted emerging and entering roost cavities at dusk) to identify colony size. Stag-watching was conducted 15 minutes before dusk and continued until no bat emerged after a period of 10 minutes since last bat emergence. The number of bats that emerged and returned to the roost were counted during that period with bats returning to the roost deducted from the number of emerged bats to obtain an estimate of colony size. The study was carried out with approval from Southern Cross University Animal Care and Ethics Committee under permit 15/12 and the Forestry Corporation of NSW under permit HF54617.

#### Roost tree variables

For each roost tree, we recorded: tree species (not further considered in analysis), tree height, tree cavity abundance with entrances > 20 mm following Threlfall et al. [31], roost tree diameter at breast height (DBH), tree senescent class ranging from 1 to 8, with class 1 representing trees that contain no first or second order dead branches and class 8 being dead tree stumps following Gibbons et al. [43], canopy percentage foliage cover, habitat type (i.e. riparian zone, logged areas (divided into < 10 years old and ≥ 10 years old regrowth) and non-riparian mature forest), distance to nearest stream line, and distance to nearest logging area or retained buffer.

### Measuring available trees

Local available cavity-bearing tree density was determined using the point-quarter method [44] by locating the nearest four “bat roost available trees” (> 10 cm in DBH, > 1.5 m tall and with a cavity entrance of > 1.5 cm) surrounding identified roost trees. The same tree variables were recorded as for identified roost trees. Where available trees contained more than one cavity, the cavity with the smallest entrance was recorded. The distance to the nearest roost available tree in each quadrant was used to calculate the local density of potential roost trees.

Tree availability was also sampled across the landscape. Three different forest zones were sampled: within the logged area (logging activity < 10 years old), within mature forest (estimated to have not been logged for the last 50 years) and within riparian buffers surrounded by recent harvesting. Within each zone, a 200 m transect was located randomly. At every 20 m point along the transect, the closest tree (> 10 cm DBH and > 1.5 m tall) within each quadrant was measured and presence/absence of a cavity was recorded. The transects were replicated four times for each zone and were at least 500 m apart. The landscape tree data were used to calculate available roost tree density in each habitat zone [44]. To obtain data within mature forest, transects were placed in the same forest type within the nearby Murramarang National Park (located about 5 km to the east of the study site).

### Statistical analysis

A Canonical Analysis of Principal coordinates (CAP) was conducted using PRIMER 6 (PRIMER-E Ltd, Plymouth, UK) to explore the relationship between roost tree variables (canopy cover, DBH, tree height, cavity abundance, tree senescence and distance to stream line) to that of locally available cavity-bearing trees directly surrounding the roost trees. The data were standardised and log transformed (Log(X+1)) for the analysis and a Euclidean distance dissimilarity matrix was used. Statistical comparisons of a number of roost selection criteria were conducted using t-tests (roost tree DBH and height), Mann–Whitney U test (roost tree senescence), one-way ANOVA (roost spatial distribution) and chi-square goodness-of-fit test (live and dead tree selection). The Fisher’s exact test was used instead of the chi-square test in instances where at least one of the expected frequencies was less than five.

To assess whether bats selected maternity roosts within certain logging histories or exclusion zones, we compared availability and use within each bat species’ predicted roost range following Neu et al. [45], using Bailey’s confidence interval to identify habitat selection (see [46,47]). The Bailey’s interval was calculated using the Resource Selection Analysis Software [48]. The predicted roost range for *V*. *vulturnus* was estimated to be a 1.5 km radius surrounding the trap sites based on the maximum distance travelled between the trap site and furthest recorded roost tree (1,402 m) and 3 km radius for the larger *S*. *orion* (max. distance travelled between trap and furthest roost: 1,880 m) and *N*. *gouldi* (max. distance: 2,325 m). The study area within the 1.5 km and 3 km radii comprised areas of private properties (mean 5.4 ha for 1.5 km radius and 297.4 ha for 3 km radius across the three trap sites). Given the range of habitat types within this area, ranging from cleared land to various stages of regrowth forest, and that none of the radio-tracked bats roosted within this land type, private land was not included in the analysis. No habitat analysis was conducted for *S*. *orion* due to the low sample size. To describe the proportion of habitat type and logging history available across the broader landscape, a 10 km radius was used (see *“study area”* section above).

## Results

A total of 25 lactating *V*. *vulturnus*, seven lactating *N*. *gouldi* and six lactating *S*. *orion* females were tracked. The mean tracking period per individual was 5.9 ± 0.52 days. Overall, 16 maternity roost trees were located for *V*. *vulturnus*, 15 for *N*. *gouldi* and six for *S*. *orion* (excluding one *S*. *orion* roost location where the roost tree could not be confidently identified).

### Maternity colony size and composition

Stag-watch counts estimated mean maternity roost colony size to be 29 ± 3.3 for *V*. *vulturnus* (n = 14, excluding three stag-watches that included volant young emergence), 14 ± 1.6 for *N*. *gouldi* (n = 9) and 48 for *S*. *orion* (n = 1). Not all lactating females of a species that were caught at the same trap site and during the same tracking period congregated in the same maternity colony, i.e. of the 13 *V*. *vulturnus* tracked at “Site 1”, four separate maternity colonies were identified and at “Site 3”, the 12 *V*. *vulturnus* females tracked were from at least three different maternity colonies. No roost switching was observed between the colonies that would suggest sub-group formation across different roosts, nor fission-fusion interactions [28,49,50]. *Vespadelus vulturnus* colonies surrounding the same trap sites were separated by a minimum of 71 m between roosts (mean 429 ± 39 m).

Three lactating *S*. *orion* females were trapped at the same site and the same tracking period with two of the three females found roosting together on two occasions in two differing group compositions of two over the four day overlapping tracking period. That is, on 12 December female F33 was roosting with F32 while female F12 was roosting elsewhere, whereas on the following three days, F33 joined F12 in her roost whilst F32 roosted elsewhere, indicating some maternity colony fission-fusion interaction. The two roosts surrounding the F12 roost during the overlapping tracking period were 133 m and 1,412 m away. No more than one lactating *N*. *gouldi* was successfully radio-tracked during the same period from the same trap site and thus, colony cohesion of this species could not be documented.

### Maternity roost range and movements

The roosts of *N*. *gouldi* were predominantly in the same riparian catchment in which they were caught. However, one colony roosted in a riparian gully for the first four days of tracking and then used a ‘ridge to headwater’ buffer to travel across the ridge (roosting near the ridge top on the fifth day) to roost in the neighbouring catchment for the remaining two days (i.e. until the transmitter fell off). *Vespadelus vulturnus* and *S*. *orion* roosted predominantly in the same valley with one individual of each species traveling between catchments. Distances travelled from trap sites to the first maternity roost after release varied across individuals and species. The longest distance travelled was 1,402 m (mean: 464 ± 104 m; (n = 15) for *V*. *vulturnus*, 1,917 m (mean: 798 ± 389 m; n = 4) for *N*. *gouldi* and 775 m (mean: 458 ± 69 m; n = 6) for *S*. *orion*. The distance between trap site and first roosts did not differ significantly among species (*F* = 1.08; d.f. = 2; *P* = 0.355). The mean distances travelled between roosts were 216 ± 59 m (n = 5) for *V*. *vulturnus*, 148 ± 41 m (n = 11) for *N*. *gouldi* and 607 ± 336 m (n = 4) for *S*. *orion*, with distances being marginally significantly different among species (*F* = 3.31; d.f. = 2; *P* = 0.061).

### Roost site fidelity

*Vespadelus vulturnus* commonly displayed a high roost site fidelity. Two colonies used only one roost each for the entire duration of colony monitoring; one roost was used for 33 consecutive days, the other for 28 days. A third colony used one roost for at least 27 consecutive days and was still in use at the end of the field work, however, this colony used another roost tree for the first six days of colony tracking. The two continuously used roosts were again used the following maternity season (documented by stag-watching during one evening in December 2016). Overall, the frequency of *V*. *vulturnus* maternity roost switching was 0.2 ± 0.02 per day (based on 23 females tracked for at least three consecutive days).

*Nyctophilus gouldi* switched roosts every night without exception, whereas *S*. *orion* used the same roosts over consecutive days but switched roost more frequently than *V*. *vulturnus*. *Scotorepens orion* displayed a roost switching behaviour that suggested the use of a ‘primary’ roost that is used most days (see also “*maternity colony size and composition*” section). This is based on a *S*. *orion* female tracked over 12 days using two roosts during that time. The female used the first (‘primary’) roost (12–1) for four days followed by two days in another roost and subsequently returning back to the first roost for the remaining four days of tracking. Another female tracked for four days also used the 12–1 roost over three consecutive days. Yet another *S*. *orion* individual tracked for four days, used roost 12–1 on the first day of tracking and a subsequent roost for the remaining three days. Roost re-use was not documented by *N*. *gouldi* and *V*. *vulturnus* once the roost was abandoned for a day.

### Roost tree characteristics

#### Dead and live roost trees

Dead trees made up 50% (eight trees) of *V*. *vulturnus* roosts, 47% (seven trees) of *N*. *gouldi* roosts and none for *S*. *orion* roosts. The frequency of use between dead vs live roost trees did not differ significantly for *V*. *vulturnus* (d.f. = 1; χ^2^ = 0; P = 0.718) or *N*. *gouldi* (d.f. = 1; χ^2^ = 0.13; P = 0.718) but was significantly different for *S*. *orion*, which used live trees exclusively (Fisher exact test: p = 0.002). When dead and live roost trees were compared to local available live and dead cavity-bearing trees, the frequency of dead tree use was significantly greater for *V*. *vulturnus* (Fisher exact test: p = 0.003) and *N*. *gouldi* (Fisher exact test: p = 0.007), but not for *S*. *orion* (Fisher exact test: p = 0.593).

#### Roost tree DBH and height

The maternity roost tree DBH ranged from 15 to 150 cm for *V*. *vulturnus* (mean: 71.8 ± 9.1 cm), 22 to 120 cm for *N*. *gouldi* (52.9 ± 6.5 cm) and 79 to 170 cm for *S*. *orion* (115 ± 13.6 cm). On average, dead maternity roost trees (species combined) had a significantly smaller DBH (55.0 ± 9.1 cm) than that of live roost trees (80.0 ± 7.8 cm) (t = -2.07, d.f. = 30, P = 0.048). Available cavity-bearing tree data, also showed that dead trees bore cavities at a significantly lower DBH (mean: 35.4 ± 3.3 cm) to that of live trees (87.5 ± 3.8 cm) (t = -10.07; *P* = 0.001). When roost tree DBH (both live and dead trees) of the different bat species is compared to locally available cavity-bearing DBH, roost DBH was significantly smaller for *N*. *gouldi* (t = 3.59, d.f. = 24, P = 0.001), significantly larger for *S*. *orion* (t = -2.50, d.f. = 6, P = 0.046) and not significant for *V*. *vulturnus* (t = 0.79, d.f. = 20, P = 0.438).

The mean maternity roost tree height for *V*. *vulturnus* was 15.7 ± 1.7 m (n = 16), 15.5 ± 1.6 m (n = 15) for *N*. *gouldi* and 25.0 m ± 3.2 (n = 6) for *S*. *orion*. Tree height was significantly different between dead (mean 14.0 ± 1.9 m) and live roost trees (mean 19.3 ± 1.5 m) (t = -2.20, d.f. = 30, P = 0.035) for species combined. Tree height of live roost trees (species combined) was not significantly different to tree height of local available live cavity-bearing trees (t = -0.18, d.f. = 28, P = 0.862), but dead roost trees were significantly taller compared to available dead cavity-bearing trees (t = -2.79, d.f. = 16, P = 0.013). Roost tree height (both dead and live trees) compared to available cavity-bearing trees was not significantly different for species (*V*. *vulturnus*: t = 0.96, d.f. = 19, P = 0348; *N*. *gouldi*: t = 1.08, d.f. = 18, P = 0.293; *S*. *orion*: t = -2.29, d.f. = 5, P = 0.071).

#### Roost tree senescent class

Maternity roost tree senescent class 6 (dead trees with second order branches persisting) was most commonly used by *V*. *vulturnus* (mode: 6; n = 16) and *N*. *gouldi* (mode: 6; n = 15), whereas class 2 trees were most commonly used by *S*. *orion* (mode: 2; n = 6). Comparing *V*. *vulturnus* and *N*. *gouldi* roost tree senescence to *S*. *orion* roost tree senescence, the selection was significant (*V*. *vulturnus*: U = 8.5; P = 0.002; *N*. *gouldi*; U = 13.5; P = 0.011). The mode for available cavity-bearing roost senescent class was 2 (n = 120). Available cavity-bearing tree senescent class differed significantly compared to *N*. *gouldi* roost trees (*N*. *gouldi*; U = 569.0; P = 0.018), selecting trees of greater senescence, and *S*. *orion* (U = 184.5; P = 0.040), selecting roost trees of lesser senescence, but not for *V*. *vulturnus* (U = 112.5; P = 0.758).

#### Environmental correlates of roost tree preferences

**Fig 2:**
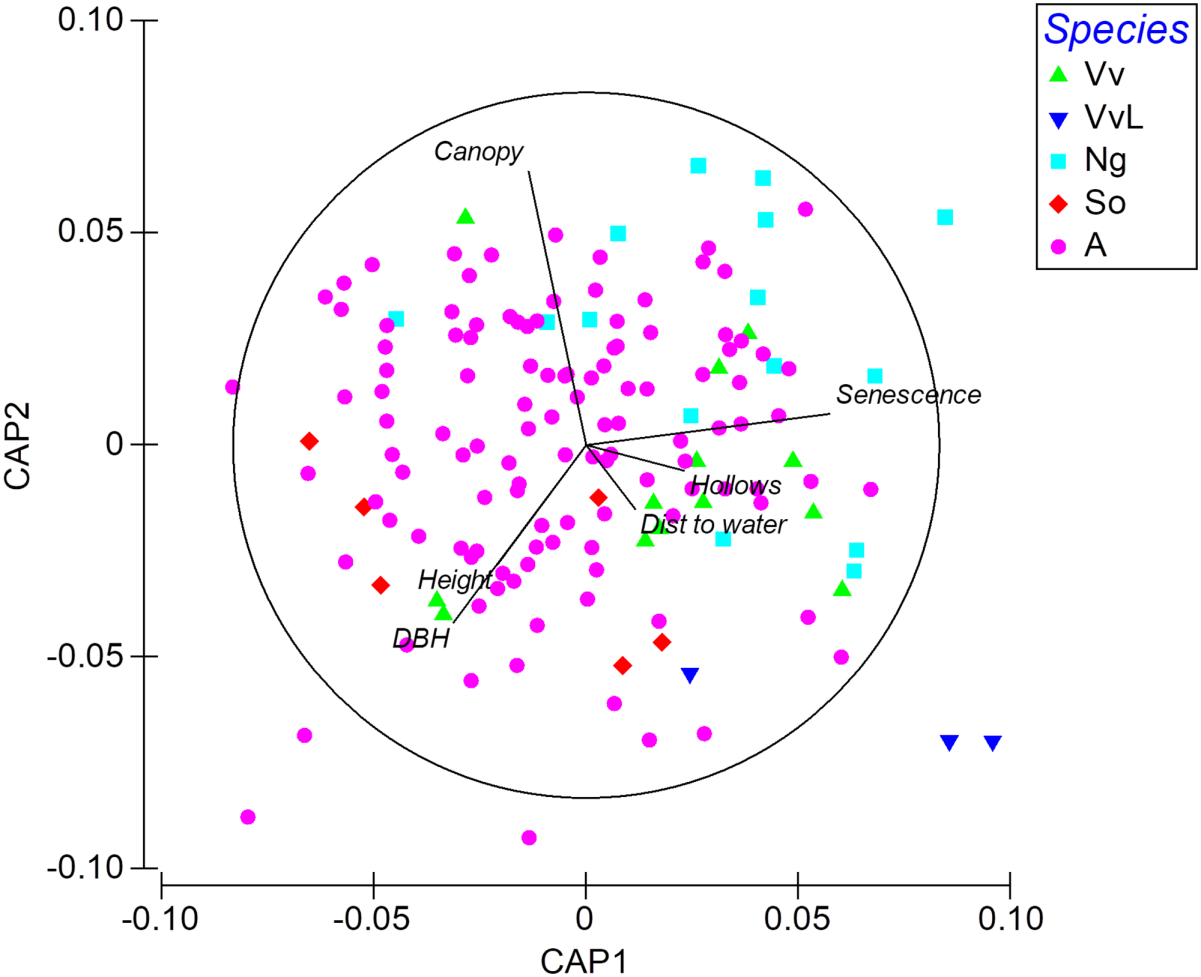
Canonical Analysis of Principal coordinates output showing available cavity-bearing tree and roost tree characteristics, using six variables. Vv = *V*. *vulturnus* roost trees; VvL = the three *V*. *vulturnus* roosts used long-term; Ng = *N*. *gouldi* roost trees; So = *S*. *orion* roost trees; A = locally available cavity-bearing trees surrounding the roosts. Dist to water = distance to stream line. Hollows = abundance of cavities within tree.

The visual examination of the CAP output (Fig 2) indicates that available cavity-bearing tree characteristics represented a range of different tree attributes and at varying distances from streams. *Nyctophilus gouldi* roost trees were associated with high tree senescence and weakly with tall trees and large DBH. For *V*. *vulturnus* roost trees, the CAP indicates an association with low canopy foliage cover, particularly for the three roosts used long-term (also remote from stream lines), whereas *S*. *orion* roost trees tended to be large trees of low senescence.

### Landscape roost selection in relation to logging

*Vespadelus vulturnus* roosted in a number of habitat types, including riparian buffers on 10 occasions (63%), recently logged compartments (less than four years since logging) on five occasions (31%) and on one occasion in forest logged > 10 years ago. All but one *N*. *gouldi* roost trees were located in riparian buffers (n = 14) with one located within an 80 m wide ‘ridge to headwater’ logging exclusion buffer. *Scotorepens orion* roost trees were found in riparian buffers on six (86%) occasions and once in a recently logged compartment (less than two years since logging) in an isolated tree.

**Fig 3:**
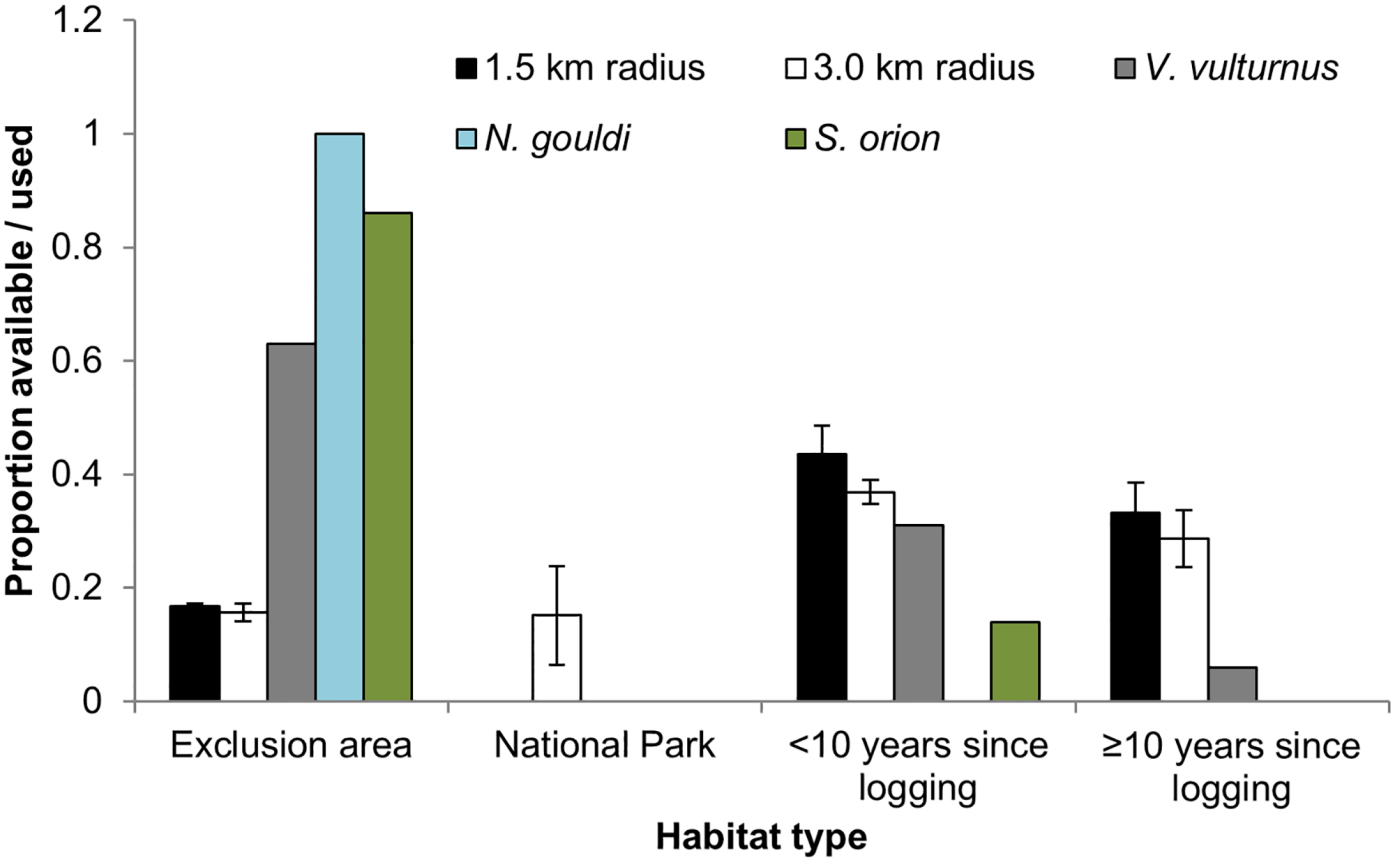
Proportion of habitat type and proportion of roost trees within habitat type in the study area (1.5 km radius surrounding trap sites for *V*. *vulturnus* and 3.0 km for *N*. *gouldi* and *S*. *orion*).

Logged areas comprised the most common habitat type surrounding the trap sites, occupying 83% within the 1.5 km radius and 81% within the 3 km radius of State Forest land. The area of recent logging (< 10 years old) made up a slightly greater proportion than that of the area with an older forest logging history for both radii. The area of logging exclusion within State Forest land made up a mean 16% within the 1.5 km radius and 19% in the 3 km radius with riparian buffers making up most of the logging exclusion area (Fig 3).

*Vespadelus vulturnus* showed a preference (G (adj.) = 18.12; P = < 0.001) for roosting in exclusion zones (i.e. riparian buffers), an avoidance for areas that experienced logging ≥ 10 years, and used roost trees within the area of < 10 years since logging in proportion to availability. *Nyctophilus gouldi* showed a strong preference (G (adj.) = 46.08; P = < 0.001) for riparian buffers and avoidance of the other habitat types (Fig 3). No analysis was undertaken for *S*. *orion* due to low sample size. However, six of seven roost trees for this species were within riparian buffers.

Cavity-bearing tree density across the landscape was densest within mature non-riparian forest (~140 trees ha^-1^), followed by riparian buffers (~82 trees ha^-1^) and recently logged areas (~1.4 trees ha^-1^) (*F* = 45.10, *P* = 0.001). At a landscape scale, this equates to ~33,860 cavity-bearing trees in our 2,058 ha 3 km radius study area (~16 cavity-bearing trees ha^-1^) within State Forest land. Calculated mean cavity-bearing tree density surrounding *V*. *vulturnus* roosts was 7.4 trees ha^-1^, of which 0.6 trees ha^-1^ were dead. The mean cavity-bearing tree density surrounding *N*. *gouldi* roosts was 12.8 trees ha^-1^, of which ~3.0 trees ha^-1^ were dead. The cavity-bearing tree density surrounding maternity roosts for both species was on average 31 times less than available in mature forest, 18 less than in riparian forest and three times greater than in logged areas. No local available cavity-bearing tree data were obtained for *S*. *orion* due to the low numbers of identified roost trees.

## Discussion

### Habitat type roost selection

A correlation between high tree cavity density and bat roosting has been documented both in Australia (e.g., [7,16]) and overseas (e.g., [13,34,35,51]). Few studies have documented flexibility of roost selection in landscapes containing low numbers of tree cavities [20–23]. Our study in recently logged forest documented that *V*. *vulturnus* maternity colonies selected roosts in both highly modified logged areas comprising young regrowth and in less disturbed riparian buffers. This use of logged areas differs to studies on two congeners. The eastern forest bat (*V*. *pumilus*) was documented to select maternity roosts in gullies excluded from logging within a 20 to 30 year old regrowth forest habitat [6]. Similarly, the southern forest bat (*V*. *regulus*) infrequently roosted in logged areas in Western Australia, although the study was conducted outside the maternity season [16].

*Nyctophilus gouldi* differed in roost habitat selection to *V*. *vulturnus* by roosting exclusively in logging exclusion zones. Maternity roosting in riparian and densely vegetated non-riparian areas has been documented previously for *N*. *gouldi* [30,31]. The preference of roosting in riparian zones may be skewed due to largely absent intact forest patches outside riparian buffers at our study area. *Scotorepens orion* roost data were limited but indicated a preference to roost in riparian zones. However, this species used emergent trees possibly because this species avoids smaller cavity-bearing trees surrounded by dense vegetation. The absence of *N*. *gouldi* and *S*. *orion* roosts in National Park land (i.e. containing areas of non-riparian mature forest) is likely due to this land type only occurring along the fringes of the 3 km radius, outside the core roost area (Fig 1).

### Roost tree characteristics

**Fig 4:**
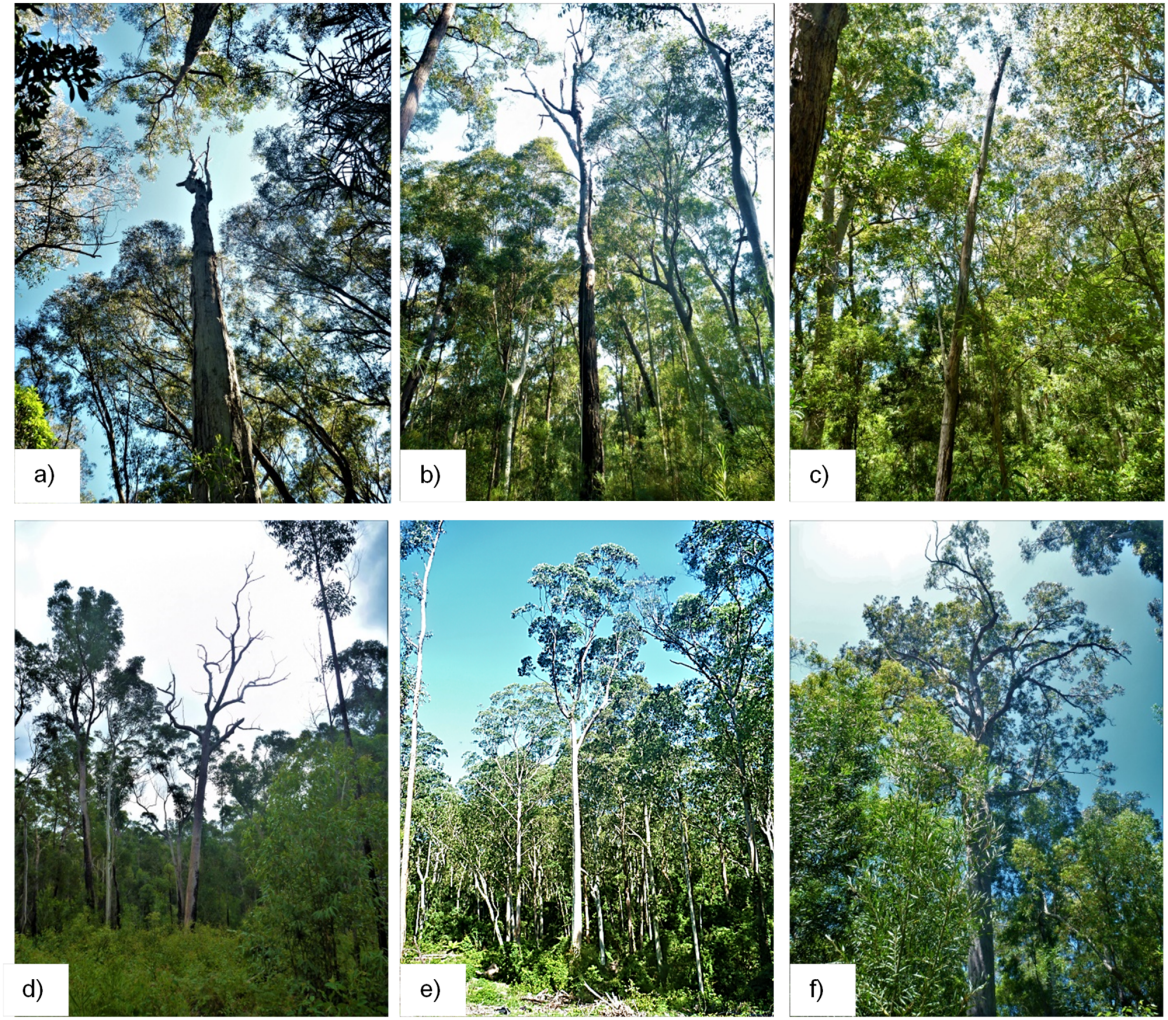
Range of roost trees used: a & b) *N*. *gouldi* dead roost trees, c) small diameter (15 cm DBH) dead *V*. *vulturnus* roost tree, d) large (1.10 m DBH) *V*. *vulturnus* long-term used dead roost tree in logged area, and e & f) large live *S*. *orion* roost tree (1.12 m and 0.86 m).

It is well established that dead trees are an important roost resource (e.g., [9,14,22,34,35,38]). In our study, both *N*. *gouldi* and *V*. *vulturnus* showed a preference for dead roost trees over live trees compared to availability, whereas *S*. *orion* roosted in tall living trees that were of low senescence (Fig 4). The preference of dead roost trees has been documented previously for *N*. *gouldi* [31] and *V*. *vulturnus* [32].

It is unclear why bats commonly display a preference for cavities in dead trees. Two hypotheses have been proposed previously: (1) entrances to dead tree cavities may be tighter, i.e. fissure-like, which facilitates predator protection, and 2) dead trees may contain less vegetation clutter in front of the cavity entrance, which facilitates easier access for bats and reducing aerial predator predation [52,53]. Other potential reasons that may influence cavity selection in dead trees are: (1) cavities in living trees, unlike cavities in dead trees, have the ability to respond to cavity causing organisms through compartmentalisation [54], thus potentially limiting internal cavity formation suitable for colony formation, and (2) fungi that cause decay in dead wood (saprophytes) may form different cavity types to that of fungi that feed on living tissue (pathogenic fungi). Further research is required to test whether microclimates differ between cavities surrounded by dead versus live xylem and the potential influence this may have on cavity selection by lactating bats.

Studies have documented the selection of large DBH trees by Australian bats (e.g., [6,9,16,53,55]), including by *N*. *gouldi* [16,30,56], as well as by tree cavity-roosting bats overseas (e.g., [36,37,57]). We found that *S*. *orion* showed a preference for large diameter and tall trees for maternity roosting consistent with the literature, whereas *N*. *gouldi* and *V*. *vulturnus* did not rely upon large diameter trees at our study site with small diameter trees, in particular dead trees, providing maternity roost resources for both specie*s*.

### Roost switching

The high roost site fidelity by *V*. *vulturnus* was unexpected given frequent roost switching is common by tree cavity-roosting bats [3,5,39]. Postulated benefits of roost switching are predator and ectoparasite avoidance, closeness of hunting grounds and switching roost due to: adverse changes in roost microclimates, interspecific competition or social interactions (e.g., [39,50,52]). A potential benefit of using the same maternity roost for an extended period may be that no energy is spent transporting non-volant young to roost sites [32,58,59]. This may be particularly important for *V*. *vulturnus* given it is one of Australia’s smallest bats (mean weight 4 g [29]). However, the long-term roosts continued to be used even after young became volant. Furthermore, lactating females of a similarly sized species, *V*. *pumilus*, regularly switched roosts [6].

It is likely that certain roost characteristics must be met to facilitate *V*. *vulturnus* long-term roosting. These may include: tight fitting cavity entrance for predator protection and to exclude large cavity-using competitor species [60,61], provision of suitable microclimates [12,62,63], roost cavity depth below entrance to allow guano accumulation [64], sufficient cavity volume for mothers to form a colony and for growing pups [8], as well as cavity height above ground (mean = 18.3 ± 3.7 m; for the three *V*. *vulturnus* long-term roosts) and non-interlocking canopies for predator protection [61,65].

It may be that logging had the effect on some *V*. *vulturnus* colonies to switch maternity roosts more frequently than they would in an undisturbed forest landscape as the likelihood of tree cavities being available to meet long-term use characteristics is reduced. Conversely, it may be that isolated retained cavity-bearing trees are conducive for long-term use if maternity roost selection is in part influenced by roost cavities being sun exposed or by roosts that provide restricted predator access through a lack of interlocking canopy cover. The CAP analysis indicated that the three *V*. *vulturnus* roosts used for prolonged periods were correlated with low canopy foliage cover which was a result of logging.

In contrast to *V*. *vulturnus*, *N*. *gouldi* switched roosts every day. Frequent roost switching has been documented in previous studies for this species [30,31], with Threlfall et al. [31] reporting that 71% of maternity roosts were switched every night. *Nyctophilus gouldi* roost switching was localised and tended to occur within the same riparian buffer, although one colony used a ‘ridge to headwater’ buffer to link up with the neighbouring catchment. The short distances travelled between roost sites (mean = 148 ± 41 m) indicate that maternity colonies have a fidelity to a specific forest area for roosting [30,31], which has been previously documented for other species [28]. This area fidelity may be due to the energy expense of carrying non-volant young to new roosts with *N gouldi* frequently having twins [66].

*Scotorepens orion* switched maternity roosts at a frequency between that of *V*. *vulturnus* and *N*. *gouldi*. The tracking suggests that this species may use primary roosts where females return after a brief absence in nearby ‘secondary’ roost trees, potentially similar to the ‘communal hubs’ described for the white-striped free-tailed bat (*Austronomous australis*) [67]. The roost switching of individuals between presumed subgroups of a larger maternity colony indicates some fission-fusion behaviour as has been described for other species [28,49,50]. We collected few data for *S*. *orion* however, and more research is required. If primary roost sites are used, identification and protection of these trees would likely be important as the loss of such trees may disrupt social interactions between individuals and roost networks [67].

### Forest management implications

The area of logging exclusion at the study site within State Forest land comprised < 20% within a 1.5. km, 3.0 km and 10 km radius surrounding the trap sites. This is below the 33% retention average in the southern region of New South Wales [68]. Furthermore, mature forest in this landscape contained an average 28 times greater cavity-bearing tree density than surrounding maternity roosts and a 100 times greater density than logged areas across the landscape, highlighting the impact past and current logging has had on cavity-bearing tree density. However, the persistence of the three species tracked at the study site that experienced intense logging over many decades indicates some resilience to logging by these species, though potentially at reduced population sizes.

The importance of dead trees for *V*. *vulturnus* and *N*. *gouldi* maternity roosts, including small diameter trees (as small as 15 cm in DBH in this study; Fig 4c), highlights the need to retain such trees. Where isolated tree retention (dispersed retention) is practiced, small dead trees are vulnerable to being lost in logged areas during a logging operation, e.g. due to under-scrubbing for machine access, tree felling, timber extraction or burning logging debris. Adopting an aggregated retention approach, where clumps of intact forest are retained, would likely be more effective in retaining smaller dead trees (see also *‘retention forestry approach’* section below).

At our study site, past and current logging exclusion zone prescriptions are skewed towards retaining cavity-bearing trees in riparian and other gully vegetation resulting in low cavity-bearing tree densities mid and upslope. The bias for gully vegetation retention is well justified given the recognised importance of this habitat for many wildlife species (e.g., [13]), including for bats (e.g., [6,31,69]). However, the dense gully vegetation cover may result in reduced access to some tree cavities for clutter sensitive bat species. Similarly, a comparable situation may occur in logged areas where isolated trees are retained with maturing (un-thinned) regrowth increasing clutter over time. In addition, the lack of cavity-bearing tree retention mid and upslope may affect cavity-roosting bat species that have different topographic roost selection requirements within a year [6,37,70] or may affect species that display a roost preference for upper slope locations [23].

#### Retention forestry approach

Our data indicate that the forest management at our study site retains adequate maternity roost habitat for the three species studied. However, in the absence of data that inform vegetation retention thresholds within a local landscape [13,24,25] and the general lack of data on species-specific maternity roost habitat requirements, including for threatened species, conservative vegetation and cavity-bearing tree retention/recruitment prescriptions are warranted. In order to preserve more diverse habitat in logged areas, including cavity-bearing trees and uncluttered vegetation, the implementation of a ‘retention forestry’ approach as defined in Lindenmayer et al. [25] should be considered. This approach should include the retention of forest patches (aggregated retention) both mid and upslope within logged areas with the patches receiving in-perpetuity protection from logging [25]. In-perpetuity protection will ensure that aspects of mature or intact forests, including cavity-bearing trees and dead trees of varying sizes and senescence, are more evenly distributed over the landscape and increases the likelihood of providing greater forest biodiversity complexity [71].

Other benefits of retaining forest patches in logged areas for bats are: (1) the retention of cavity-bearing trees across different topographic locations, (2) increased protection of small diameter dead trees from logging impact, (3) increased protection of retained trees from wind throw, and (4) the retention of a greater diversity of cavity-bearing trees and cavity characteristics, including cavities with small entrances that are not readily seen from the ground during pre-logging cavity-bearing tree surveys [72]. In addition, increasing the retention of vegetation within logged areas is considered a conservative management approach in the absence of more detailed data on species-specific roost tree selection, such as *S*. *orion* ‘primary’ roost trees and *V*. *vulturnus* ‘long-term’ roost trees that cannot be distinguished at present on the basis of commonly measured attributes.

Next to adding forest patches within logged areas, increasing the frequency at which ‘ridge to headwater’ buffers are implemented should also be considered. Such retention buffers comprise a variety of vegetation communities and would assist in increasing the conservation of intact forest areas mid and up slope, as well as connectivity between catchments. Furthermore, a greater emphasis should be given to the recruitment of cavity-bearing trees in logged areas where density is low. At our study area, current policy only requires the recruitment of cavity-bearing trees (up to 5 ha^-1^) if existing cavity-bearing trees are present. This policy has resulted in an estimated average of 1.4 recruitment cavity-bearing trees ha^-1^ retained in logged areas which is considered low. Adapting a ‘retention forestry’ approach in logged areas would ensure the protection of an increased number of cavity-bearing tree recruits.

## Supporting information

S1 File Field data. (XLSX)

S2 File GIS data for area calculations (including for Fig 3). (XLSX)

S1 Fig Canonical Analysis of Principal coordinates data. (XLSX)
